# Multiple Cues for Winged Morph Production in an Aphid Metacommunity

**DOI:** 10.1371/journal.pone.0058323

**Published:** 2013-03-05

**Authors:** Mohsen Mehrparvar, Sharon E. Zytynska, Wolfgang W. Weisser

**Affiliations:** Terrestrial Ecology, Department of Ecology and Ecosystem Management, Center for Life and Food Sciences Weihenstephan, Technische Universität München, Freising, Germany; Université Paris 13, France

## Abstract

Environmental factors can lead individuals down different developmental pathways giving rise to distinct phenotypes (phenotypic plasticity). The production of winged or unwinged morphs in aphids is an example of two alternative developmental pathways. Dispersal is paramount in aphids that often have a metapopulation structure, where local subpopulations frequently go extinct, such as the specialized aphids on tansy (*Tanacetum vulgare*). We conducted various experiments to further understand the cues involved in the production of winged dispersal morphs by the two dominant species of the tansy aphid metacommunity, *Metopeurum fuscoviride* and *Macrosiphoniella tanacetaria*. We found that the ant-tended *M. fuscoviride* produced winged individuals predominantly at the beginning of the season while the untended *M. tanacetaria* produced winged individuals throughout the season. Winged mothers of both species produced winged offspring, although in both species winged offspring were mainly produced by unwinged females. Crowding and the presence of predators, effects already known to influence wing production in other aphid species, increased the percentage of winged offspring in *M. tanacetaria,* but not in *M. fuscoviride.* We find there are also other factors (i.e. temporal effects) inducing the production of winged offspring for natural aphid populations. Our results show that the responses of each aphid species are due to multiple wing induction cues.

## Introduction

Phenotypic plasticity is the ability of a single genotype to produce more than one alternative phenotype in response to environmental conditions, or in other words the ability of a single genotype to express itself in different ways in different environments [1], [2]. In some cases, phenotypic plasticity will be expressed as several highly morphologically distinct results and this is termed polyphenism [3]. The caste system in social insects such as Hymenoptera and Isoptera is one of the most striking examples for polyphenism with morphologically distinct, but most of the time genetically identical, workers and soldiers [3], [4]. A wide variety of environmental stimuli can induce different phenotypes in organisms [1].

Aphids exhibit polyphenism, such that genetically identical individuals can potentially show different phenotypes, e.g. they can have wings or be unwinged [5]. Winged aphids are specialized for dispersal through flight, as they have a more developed sensory system, are more resistant to starvation and live longer [6], [7]. These features are assumed beneficial for locating new habitats and host plants by winged aphids in a complex environment; each aphid species feeds on a restricted range of host plants and thus the locating, landing and quick initialisation of reproduction on suitable host plants is imperative to the fitness of the aphid [8]. Winged aphids have also been shown to have reduced fecundity and longer developmental times, which is likely due to the increased energy cost of having wings [6], [9], [10].

The production of winged disperser morphs in aphids can be influenced by both genetic and environmental factors [11]. The production of winged male aphids has been shown to be under genetic control [5], [12], [13], [14]. However, the production of winged offspring from asexual mothers is likely influenced by a number of environmental factors rather than under genetic control [15], [16], [17]. In some aphid species, winged individuals are often produced at a certain times in the season; for example, winged dispersers are produced in early summer and then again at the end of the season, when winged individuals are mostly sexual morphs [18].

A considerable number of studies have addressed the environmental conditions that affect the production of winged individuals in aphids; crowding, interspecific interactions, host plant quality and abiotic factors induce winged morph production in aphids [5], [11], [13], [19], [20], [21]. Increasing the population density of an aphid colony (i.e. crowding) creates greater tactile stimulation among individuals, which can trigger wing induction [22], [23]. This increased production of winged dispersers regulates the population size of a colony on a plant, as the winged aphids move away to find new host plants. The degree of sensitivity to crowding is not the same across aphid species, and even varies between different genotypes within the same species [15], [16], [22], [23], [24]. The mere presence of natural enemies, including predators and parasitoids, is known to elicit wing induction in aphids [25], [26], [27], [28], [29], [30], [31], potentially through pseudo-crowding effects, through changes in aphid density as aphids are consumed/parasitized, or through the production of chemicals (e.g. aphid alarm pheromones) as the natural enemy moves among the plants [32], [33], [34]. It is advantageous for an aphid colony to quickly produce winged morphs when a predator is present, in order to leave a plant if the risk of predation is high. Furthermore, interspecific interactions among different aphid species that occupy the same host plant can also be a cue for enhancing production of winged morphs, likely through effects on aphid density [5], [35].

Dispersal is important for aphids in order to find new host plants, or to escape sub-optimal conditions. Many aphids exhibit a metapopulation structure with frequent extinction of small local populations. A metapopulation is defined as a set of sub-populations connected by limited dispersal, with frequent extinctions and recolonization of sub-populations [36]. In a metapopulation setting, dispersal is even more important as each plant is an ‘island of resource’ separated by unsuitable habitat that the aphids have to navigate before reaching the next suitable plant. Two specialist aphid species (*Macrosiphoniella tanacetaria* and *Metopeurum fuscoviride* (Hemiptera: Aphididae)) on tansy (*Tanacetum vulgare* (Asteraceae)) exhibit classic metapopulation structuring [37], [38], [39]. *Metopeurum fuscoviride* is tended by ants, which can protect aphids from predators and may inhibit wing production [5]. *Macrosiphoniella tanacetaria* is not ant tended. Together, they form a metacommunity [40] where dispersal and local extinctions structure the composition of local communities. It is unknown which environmental factors induce the production of winged morphs in these species.

In this paper, we follow winged morph production in two tansy aphid species to investigate the environmental cues that cause dispersal in these aphids, which is important for the understanding of the metacommunity dynamics. We follow the aphids across generations, over the season, to show that wing production changes through time. It has previously been suggested that in a species with prenatal wing determination the winged offspring will not themselves produce winged individuals, regardless of the environmental conditions [13], [18], [41], [42], and we consider maternal morph to see if this is true in our tansy-aphid system. Further, we tested the influence of increased crowding and the presence of a predator on the proportion of winged aphids.

## Materials and Methods

Potted tansy plants were grown in a greenhouse until 20–25 cm in height prior to experimental use. The greenhouse conditions were ∼25°C during the day and ∼20°C at night with a 16h light 8h dark regime. As *M. fuscoviride* is an obligate myrmecophilous aphid, the ant species *Lasius niger* had access to its colonies during all experiments. Aphid wing-dimorphism phenotype identification was performed only on 4^th^ instar nymphs or adults; winged 4^th^ instar nymphs of aphids can easily be recognized from wingless ones by the presence of wing buds on their thorax. To exclude effects of genetic variation in the aphids, we used different genetic lines of aphids for each experimental replicate. Meteorological data were obtained from a weather station in the Jena Experiment (4 km away from the experimental site).

### 1. Seasonal Life Cycle and Time of Appearance of Winged Individuals of *M. tanacetaria* and *M. fuscoviride*

To follow the production of winged morphs over all generations throughout the season, colonies of tansy aphids were located early in the season, i.e. beginning of April when first-generation individuals (fundatrices) hatched from eggs. In 2010, second-generation aphids were collected from the field and used in the experiment. In 2011, fundatrices were collected and then placed on experimental plants.

In 2010, the generations of *M. tanacetaria* (MA) aphids were followed and the appearance of winged morphs was observed. On 12^th^ May 2010, seven potted tansy plants were placed inside cages (28×28×120 cm), to prevent infestation by non-experimental aphids and natural enemies. The cages consisted of an aluminium frame, with thick polyethylene sheets forming the top and two sides of each cage, while the two other sides were covered by fine mesh to allow airflow. The open under-side was placed inside a plastic box that had its outer walls painted with Fluon (Fluoropolymer Dispersion, Whitford GmbH, Germany), a product which on drying creates a slick barrier, thus excluding ants and other arthropods. These cages were placed outside of the Institute of Ecology, Jena, Germany, and checked every two or three days for watering and counting aphids. Two apterous (unwinged) viviparous females of MA (2^nd^ generation, collected from a field site) were placed on the plants and allowed to reproduce for two to three days. The adults were then removed. When the nymphs developed into 4^th^ instar or adults (and before they started to produce offspring) the numbers of winged and unwinged individuals were counted. Then, all individuals except two unwinged adults were removed and these remaining two aphids were allowed to reproduce for two or three days, resulting in between 5 and 20 offspring. The two adults were removed and the offspring allowed to further develop on the plant. This was repeated until the end of the season, when all individuals were of the sexual morph. This procedure allowed a clear separation of generations and ensured that crowding was minimal.

In 2011, the generations of both *M. tanacetaria* (MA) and *M. fuscoviride* (ME), and the number of winged/unwinged aphids, were observed throughout the season until colonies were exclusively constituted of sexuals. Ten potted tansy plants were selected and placed inside aluminium-frame cages (as previously described). These cages were placed outside in the botanical garden of Jena, Germany. ME is obligatory ant-attended and therefore the bottom of each cage was not placed in a plastic tray to encourage natural ant attendance. There were numerous ant nests in the vicinity of experimental place, therefore aphid colonies were easily attended by ants. Two fundatrices were then placed on each plant, and allowed to reproduce for two to three days. The same method for generation separation was used as in 2010 and the presence of ants on plants in ME cages was checked every two days.

### 2. Winged Offspring Production by Winged and Unwinged Mothers

In order to determine if winged mothers of MA produce winged offspring, we collected 15 winged individuals, in June 2011, from different un-crowded natural colonies in Jena, Germany and put them individually on a freshly detached tansy leaf, which was placed upside down onto a plastic petri dish (15 cm in diameter) containing a thin layer of 0.7% Agar gel (experiment 2.1). The dishes were maintained in a climate chamber with temperature held at 20±2°C with 16h light, 8h dark regime. The winged aphids were allowed to reproduce for three days and then they were removed. The produced offspring were allowed to develop into either 4^th^ instar or adult stage and then the numbers of winged and unwinged were counted.

Additional experiments were made with MA, to see whether unwinged mothers produce more winged offspring than winged mothers. Here, two experiments were performed, one in the climate chamber using petri dishes (experiment 2.2); and another in the greenhouse using potted plants (experiment 2.3). For the climate chamber experiment (2.2), 20 unwinged MA were used and this was run concurrently with the previous winged mother experiment (2.1), using the same experimental set-up in order to allow for comparison. For the greenhouse experiment (experiment 2.3), 15 unwinged MA were collected from different tansy plants in Jena, Germany. These adults were placed on 15 potted tansy plants (one aphid per plant, as 15 lines) in the greenhouse. In order to prevent the escape of aphids, each plant was placed in a Plexiglas cage (35×35×90 cm). These cages have three sides, plus the top and bottom, of Plexiglas, and the front covered in a fine mesh to allow airflow. The adults aphids were allowed to reproduce for three days, then all removed except five nymphs that remained on each plant. When the nymphs reached the adult stage, they were allowed to reproduce for three days and then they were removed. In this step the number of nymphs which remained on each plant was between 15–20 individuals, in order to maintain low densities of aphids. This rearing process was repeated until the nymphs of 4^th^ generation molted to 4^th^ nymphal instar. At this time, five unwinged and five winged nymphs from each plant were placed on two new plants separately and allowed to become adults and reproduce for three days. Afterwards, the adults were removed and nymphs were allowed to become 4^th^ nymphal instar or adult in both winged and unwinged mother treatments, then they were collected and frozen for later counting and examining of the phenotype. This experiment was conducted in October 2010.

In 2012, another experiment was conducted (experiment 2.4) with 40 winged ME aphids in order to determine if winged mothers produce winged offspring. The experimental procedure was similar to experiment 2.1 with the exception that the ME winged aphids were collected from tansy around Freising, Germany and the aphids were maintained at room temperature (20–25°C) with natural light availability (June) and additional access from an ant colony.

### 3. Effect of Crowding

In 2012, 20 unwinged adults of each MA and ME were collected from different colonies in the field and reared individually on tansy plants (as 20 lines) in low colony densities for two generations. Nineteen lines of MA and 15 lines of ME were used for the experiment (experiment 3). There were two treatments for each line, crowding and control treatments. Two unwinged females from each line were randomly selected for the experiment (one per treatment). For the crowding treatment, one adult was placed together with another 30 aphids (4^th^ nymphal instars and adults) from the same line in a small plastic vial (1.5 ml) to enhance crowding conditions and for the control treatment the single adult was placed in another vial. After 24 h, the adults from each of the control and crowded treatments were placed individually on a new tansy plant. The aphids were allowed to reproduce for three days, after which the adults were removed and offspring allowed to develop into 4^th^ nymphal instar or adults. Then the numbers of winged and unwinged morphs were counted.

### 4. Effect of Predators on Wing Induction

In 2011, 15 unwinged adults from each MA and ME were collected on tansy plants in the botanical garden of Jena, Germany. Lacewing larvae, *Chrysoperla carnea* (Neuroptera: Chrysopidae) (obtained as eggs from a commercial supplier (Katz Biotech Services, Welzheim, Germany)), were used as the predator. Newly hatched larvae were reared in plastic vials (5 cm in diameter and 10 cm height) individually and were fed *ad libitum* with a mixed diet of MA and ME nymphs until they reached the 2^nd^ larval stage when they were used for the experiment. The experiment (experiment 4) was conducted using Plexiglas cages in a greenhouse (see experiment 2.3), with access by ants for the ME aphids.

To minimize maternal effect, offspring from the same mother (clonal line) were exposed to different treatments. Fifteen adults of each aphid species were placed on 15 tansy plants as 15 lines (see Fig. S1). Then these 1^st^ generation adults were allowed to give birth to offspring for two days and after that all but six nymphs were removed. These nymphs (2^nd^ generation) were allowed to grow until they reached adulthood, then three of them were transferred to a new plant where they were allowed to reproduce for a further two days. Then, all but ten nymphs (3^rd^ generation) were removed. When these nymphs developed to adults, five from each line were transferred to a new plant (i.e. five adults per plant). These adults were allowed to reproduce for two days and then they were removed from the plant together with all but 25 nymphs (4^th^ generation) remaining per line. When the 4^th^ generation nymphs reached the late 4^th^ instar, 20 individuals from each plant were split into two groups of 10 and were transferred separately to two new plants (predator treatment and control treatment). In total, 15 different lines of each aphid species were established with one plant per line for each treatment (predator and control), after the 4^th^ generation (30 plants total) (Fig. S1); each line was used as a replicate.

One 2^nd^ instar lacewing larva was released on each predator-treatment plant and allowed to feed on the aphids for three days, after which it was removed. This represented the ‘*first three-day period’*. The remaining adult aphids were counted on both treatment and control plants and transferred to new plants to continue the experiment to the ‘*second three-day period’*. This second period was used since determination of offspring phenotype occurs some time before birth, which means that the production of winged morphs can be a delayed response. Here, another 2^nd^ instar lacewing larva was released onto the predator-treatment plants, and left for three days. After the second three-day period the remaining adult aphids were counted and removed from all plants. Any offspring produced in both periods were allowed to develop to the 4^th^ nymphal instar or adults, in the absence of predators, and the number of winged and unwinged aphids were counted.

### Statistics

The results are presented as mean ± standard error. In all experiments, to compare the proportion of winged/unwinged offspring between different treatments the *cbind* function in R (R version 2.14.0, 2011) was used and binomial Generalized Linear Models (GLM) with log link function were performed.

In the crowding experiment (experiment 3), the numbers of winged individuals of MA for control treatment were all zero and therefore there was no variation in the data, so we used the non-parametric Wilcoxon Signed Ranks Test to compare the proportion of winged offspring in crowding and control treatments. For the ME crowding experiment, a GLM using binomial distribution with log link function was performed. To compare the number of produced offspring by MA and ME in crowding and control treatments, GLM using negative binomial distribution with log link function was used.

In the wing induction by predators experiment (experiment 4), to compare the number of surviving adults and total number of offspring at the end of each experimental period in control and predator treatments, a paired t-test was used. In the case that data were not normally distributed a square-root transformation was performed to normalize the data. If data were not normalized by transformation a non parametric test, Wilcoxon Signed Ranks Test, was used. To compare the proportion of winged offspring in the predator and control treatments, a GLM using binomial or quasibinomial (when overdispersed) distribution with log link function was employed. A logistic regression, using the *cbind* function in R, was used to investigate the relationship between total number of offspring in the end of each experimental part and number of winged offspring. For all paired t-tests and Wilcoxon Signed Ranks Tests, the software package IBM SPSS Statistics version 19 was used.

### Ethics Statement

No specific permits were required for the described field studies. All field locations belonged to either Friedrich-Schiller-University of Jena or Technische Universität München, and no special permission was required to work in these areas. We confirm that none of the species used were endangered or protected.

## Results

### 1. Seasonal Life Cycle and Time of Appearance of Winged Individuals

#### Macrosiphoniella tanacetaria

**Figure 1:**
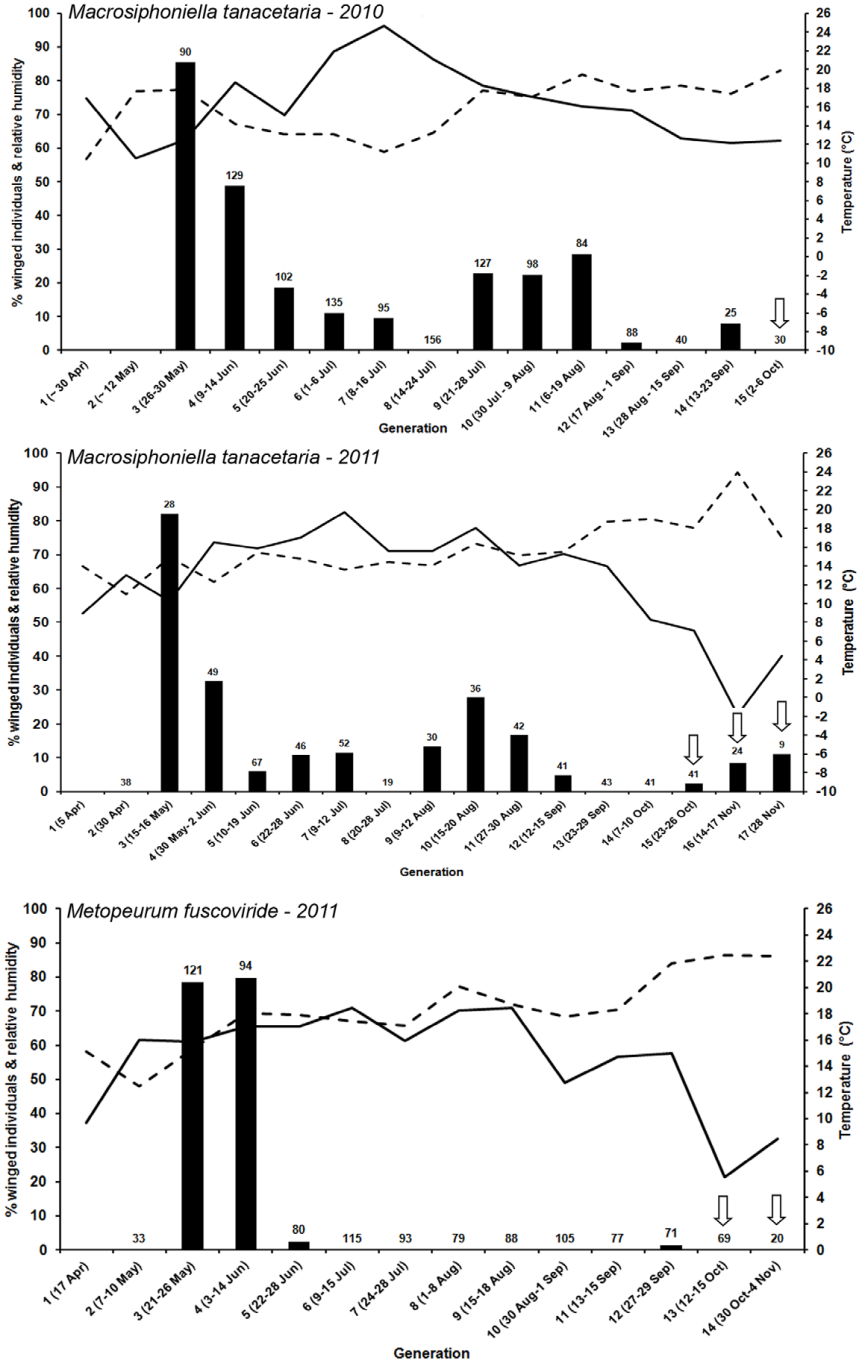
Percentage of winged individuals of *Macrosiphoniella tanacetaria* and *Metopeurum fuscoviride* in different generations. The production of winged individuals of *M. tanacetaria* (during the years 2010 and 2011) and *M. fuscoviride* (in the year 2011) during different generations was monitored and the time of appearance of sexual morphs were recorded. The arrows show generations where sexual forms were observed. The total numbers of offspring on which the winged individual percentage was based are shown on the top of each bar. The ambient temperature and humidity during the season is also showed on the graph. Solid lines show temperature and dashed lines show humidity.

On 30^th^ April 2010, the first MA colonies were observed in the botanical garden of Jena, Germany. These colonies consisted of about 20 nymphs and one fundatrix (1^st^ generation). In the experiment, there were no winged aphids observed until 26^th^ May 2010 when the third generation had been produced. In the third generation, 85.6% of the aphids were winged dispersal morphs. The proportion of winged individuals declined through June and July (to 0% in the 8^th^ generation) with a small peak during late July-August when 22.8–28.6% of offspring produced in generations 9–11 were winged (Fig. 1). The sexual individuals, apterous oviparous females, were observed in the first week of October (Fig. 1; Fig. S2). The experiment ended when all individuals were sexual females. No males were observed within the experimental populations. Fifteen generations of MA occurred in 2010.

In 2011, MA fundatrix adults were found on 5^th^ April in the botanical garden of Jena, Germany. In the experiment, there were no winged individuals during the 1^st^ and 2^nd^ generations but, as in 2010, in the 3^rd^ generation winged individuals formed the majority of the individuals (82.1%) (Fig. 1). Again, a decrease in percentage of winged individuals was observed and at the end of July (8^th^ generation) there were no winged offspring. As in 2010, there was an increase in the number of winged individuals during generations 9–11 in mid-end August (Fig. 1). The first sexual forms appeared in the 15^th^ generation, at the end of October, but asexual females were present until the 17^th^ generation in the end of November when all individuals were sexual. Thus, while some 14^th^ generation females already produced sexual offspring (7–10^th^ October), others only produced sexual offspring in the 16^th^ generation. In the 15^th^ and 17^th^ generations there was only one winged individual, which was male, while in the 16^th^ generation there were two winged individuals, one male and one viviparous female. In 2011, 15–17 generations of MA occurred.

#### Metopeurum fuscoviride

Fundatrix individuals of ME were found on 17^th^ April 2011 in a site near the Institute of Ecology, Jena, Germany. The winged individuals of ME occurred only in the 3^rd^ to 5^th^ generations (Fig. 1). The percentage of winged individuals in the 3^rd^ and 4^th^ generations was high (78.5 and 79.8% respectively) but in the 5^th^ generation it was very low (2.5%) (Fig. 1). After the 5^th^ generation there were no winged individuals produced with an exception of one winged individual that was found in one of the experimental populations at the end of September. The sexual forms occurred in the 13^th^ and 14^th^ generations (mid-October until early-November). As the sexual forms of ME (males and females) are wingless, there were no winged individuals in the end of season. ME had 13–14 generations in 2011.

### 2. Winged Offspring Production by Winged and Unwinged Mothers

Our experiments showed that winged mothers of both MA and ME are able to produce winged offspring. For MA, the mean percentage of winged offspring produced by winged mothers in the climate chamber (experiment 2.1) was low at 0.30±0.3% (overall 0.34% of offspring were winged, 1 out of 296). In the greenhouse (experiment 2.3) the percentage of winged offspring produced by winged mothers was higher 14.21±4.7% (overall 13.46% of offspring were winged, 63 out of 468); however, due to the different experimental design these results are not directly comparable. For ME in the laboratory (experiment 2.4) the proportion of winged offspring among winged mothers was 9.38±2.1% (overall 8.11% of offspring were winged, 27 out of 333). The percentages of MA and ME winged mothers which produced both morphs were 3.7% and 50% respectively, all others only produced unwinged offspring. No female produced only winged offspring.

**Figure 2:**
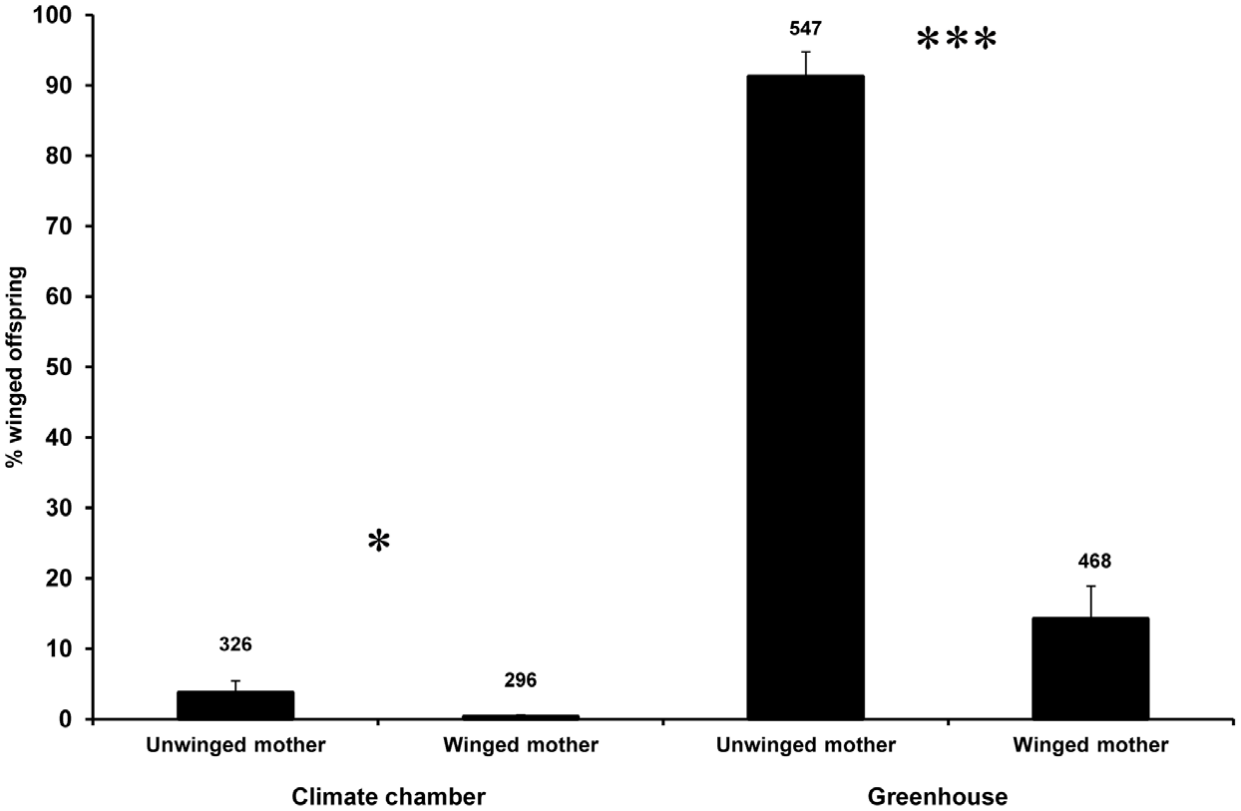
Percentage of produced winged offspring by unwinged and winged mothers of *Macrosiphoniella tanacetaria*. The percentage of winged morphs produced by unwinged and winged mothers of *M. tanacetaria* in the climate chamber (exp. 2.1 and 2.2) and greenhouse (exp. 2.3) experiments were compared. The total numbers of offspring on which the winged offspring percentage was based are shown on the top of each bar. The bars show mean±SE. *(*P*<0.05) and ***(*P*<0.001) indicate statistically significant difference between experimental treatments.

In the climate chamber experiment (experiment 2.2), unwinged mothers of MA produced 3.72±1.8% winged offspring (overall 3.68% of offspring were winged, 12 out of 326). In comparison with the number of winged offspring produced by winged mothers (experiment 2.1, also in the climate chamber and conducted at the same time), unwinged mothers produced significantly more winged offspring (binomial GLM, *Z_1, 33_* = −2.32, *P = *0.020) (Fig. 2). The percentage of MA unwinged mothers that produced both morphs was 20.5%, compared to 3.7% by winged mothers.

In the greenhouse experiment (experiment 2.3), the percentage of winged offspring produced by MA unwinged mothers was significantly higher, 6.4 times, than the percentage of winged offspring produced by winged mothers (quasibinomial GLM, *t_1,_*_28_ = −7.322, *P*<0.001) (Fig. 2). Here, 10 out of 15 plants with winged mothers had winged offspring.

### 3. Effect of Crowding

#### Macrosiphoniella tanacetaria

There was no winged offspring in the control (0.0%) while in the crowding treatment the mean percentage of winged offspring was 11.24±2.7% with 11 lines out of 19 producing winged morphs. Thus, crowding caused a statistically significant increase in production of winged morphs in MA (Wilcoxon Signed Ranks Test, *Z* = −2.941, *P* = 0.003). The number of produced offspring was affected by crowding treatment (negative binomial GLM, χ^2^ = 6.778, df = 1, *P* = 0.009) such that there were fewer aphids in the crowding treatment (363 offspring) than the control (539 offspring).

#### Metopeurum fuscoviride

The mean percentage of winged offspring produced in the control and crowding treatments were 2.38±1.1% and 2.71±1.2% respectively; crowding had no effect on the production of winged morphs (binomial GLM, *Z_1,28_* = 0.370, *P = *0.711). The number of produced offspring did not differ between crowding and control treatments (negative binomial GLM, χ^2^ = 0.191, df = 1, *P* = 0.662).

### 4. Effect of Predators on Wing Induction

#### Macrosiphoniella tanacetaria

In both experimental periods the number of surviving adults of MA was significantly lower in the predator treatment than the control (first three-day period: Wilcoxon Signed Ranks Test (data not normally distributed), *Z* = −2.981, *P* = 0.003; second three-day period: paired t-test, *t*_9_ = 4.95, *P*<0.001) (Table 1). In the first three-day period 70% and in the second three-day period 53% of adults were eaten by predators. The number of offspring was lower in the predator treatment than in the control in both experimental periods (Table 1); this was significant in the first three-day period (paired t-test, *t*_14_ = 2.628, *P = *0.02) but not in the second three-day period (paired t-test, *t*_9_ = 1.428, *P = *0.187). The reduction in adult and offspring number in the predator treatment shows that predators were actively preying on aphids in the respective replicates.

**Table 1:** Number of surviving adults and number of offspring of *Macrosiphoniella tanacetaria* and *Metopeurum fuscoviride* in the first and second three-day period under control and predator treatments.

Species	First three-day period		Second three-day period	
	Control	Predator	Control	Predator
*Macrosiphoniella tanacetaria*				
Number of surviving adults	7.8±0.9	3.0±0.8	5.2±0.8	1.9±0.6
Number of offspring	50.9±6.2	29.9±5.2	41.3±8.5	25.4±5.0
*Metopeurum fuscoviride*				
Number of surviving adults	9.9±0.1	9.5±0.3	9.5±0.2	9.1±0.3
Number of offspring	51.2±1.8	45.5±2.4	42.7±1.4	43.9±1.0

Values are mean±SE.

The flow chart shows the experimental design for one aphid line and was the same for all aphid lines.

The sexual morphs of this aphid species, produced in the autumn, lay overwintering eggs after mating.

**Figure 3:**
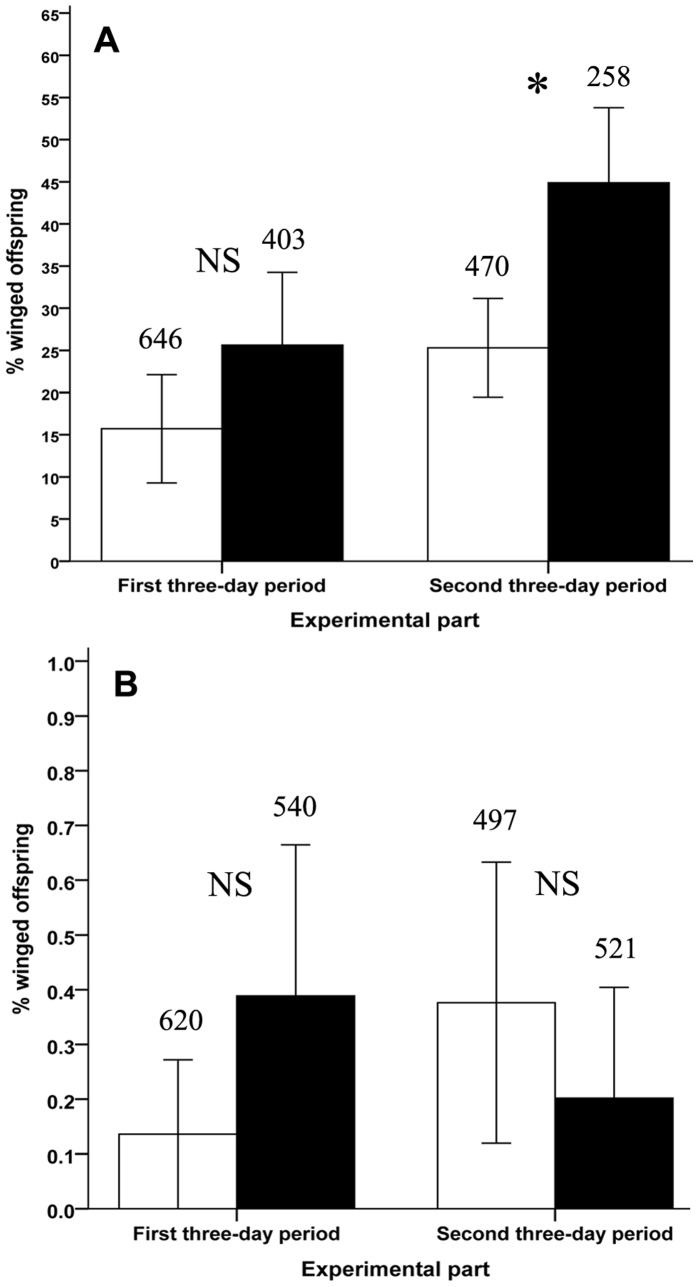
Percentage of produced winged offspring of *Macrosiphoniella tanacetaria* and *Metopeurum fuscoviride* in the presence of predators. Production of winged morphs in *M. tanacetaria* (A) and *M. fuscoviride* (B) as a function of the presence of a predator in the colony. White columns: control treatment, black columns: predator treatment. The total numbers of offspring produced in each treatment are shown on the top of each bar. The bars show mean±SE. *(*P*<0.05) indicates statistically significant difference between experimental treatments.

To consider the proportion of winged offspring produced we analysed the data together, with time period as a factor in the analysis. The presence of a predator significantly increased the percentage of winged individuals among the offspring (quasibinomial GLM, *F*_1,50_ = 6.218, *P = *0.016) so that the percentage of winged offspring was higher in the predator treatment than control (Fig. 3A). The percentage of produced winged offspring in the second three-day period was significantly higher than the first three-day period (quasibinomial GLM, *F*_1,51_ = 4.467, *P = *0.039). In the first three-day period, there was no significant relationship between the total number of offspring and the percentage of winged offspring (logistic regression, *t*_1,27_ = 0.583, *P* = 0.564). In the second three-day period the percentage of winged offspring was dependent on the number of offspring (logistic regression, *t*_1,22_ = 2.362, *P* = 0.028). This means that the number of offspring on the plant (crowding) possibly had an effect on the production of winged individuals.

#### Metopeurum fuscoviride

Contradictory with MA, predators had no effect on the number of surviving adults (first three-day period: Wilcoxon Signed Ranks Test, *Z* = −1.656, *P = *0.098; second three-day period: Wilcoxon Signed Ranks Test, *Z* = −0.998, *P = *0.318) (Table 1). In the first and second experimental periods 5.33% and 3.52% of the adults were eaten by predators, respectively. The total number of offspring in both experimental periods was similar between the predator and control treatments; however, there was a marginally significant effect of more offspring being produced in the control than the predator treatment in the first three-day period (first three-day period: paired t-test, *t*_14_ = 2.067, *P = *0.058), with no effect in the second three-day period (paired t-test, *t*_14_ = −0.648, *P = *0.528) (Table 1). These show that predators were active but because of the guarding action of the attending ants predators were not able to prey considerably on the ME.

The presence of predators had no effect on the production of winged individuals of ME in both experimental periods (binomial GLM, Predator: *Z*_1,57_ = 0.455, *P* = 0.649; Period: *Z*_1,57_ = −0.227, *P* = 0.821) (Fig. 3B). There was a positive relationship between the total number of offspring and the percentage of winged morphs among the offspring produced in the first three-day period (logistic regression, *Z*_1,28_ = 2.207, *P = *0.027) while in the second three-day period there was not (logistic regression, *Z*_1,28_ = −0.202, *P = *0.840). This means that in the second three-day period the number of offspring on the plant (crowding) had no extra effect on the production of winged individuals.

## Discussion

Our results revealed that these two specialized tansy aphids, MA and ME, differ in their responses to the environmental cues which normally trigger wing induction in aphids. In MA, seasonal/generational timing, mother morph, crowding and predators were found to have effects on the production of winged offspring. In the ant-tended species, ME, the production of winged morphs was affected by seasonal/generational timing but not by crowding or predators presence.

### Seasonal Life Cycle and Time of Appearance of Winged Individuals

In 2010, we observed MA fundatrices (the stem mothers, which hatch from the overwintering eggs) at the end of April, suggesting that the eggs hatched before the middle of April. In 2011, MA fundatrices appeared two weeks earlier, in early April, suggesting that the eggs hatched in mid-March. Thus, the first appearance of MA differed in the two years of the study, and indicates that egg hatching and emergence of the first generation may be somewhat dependent on abiotic factors, e.g. temperature (see Fig. 1). Consistent with Hille Ris Lambers [13], we found no winged offspring in the first two generations.

The number of winged individuals for both MA and ME was high in the first half of the season, at the end of May and June. This coincides with an increase in vegetative growth of tansy plants, and as such would present the optimal time for aphid dispersal to new host plants. From following the seasonal pattern of wing production of MA, we found that this aphid species produced winged individuals throughout the whole season and therefore dispersal by flight is possible at any time. In both years, MA aphids did not produce any winged individuals in the 8^th^ generation, which could be due to temporal effects on wing production; we found the proportion of winged offspring steadily fell from generation three to seven, but then increased again for generations nine through eleven for both study years (Fig. 1). In contrast, ME aphids produced the vast majority of winged morphs at the beginning of the season and this means their dispersal, followed by population expansion, occurs as one single major event. Therefore, for ME aphids, dispersal in the late season probably plays only a minor role in their population dynamics.

Variation in the production of winged individuals through the season is common across many aphid species but this has mostly been attributed to day length [18]. Here, we show that, consistently across the two study years, generation eight of the MA aphids produced no winged morphs whereas winged morphs were produced in the previous and the next generation. The difference in day length across these generations is minimal, especially as they occur across the summer solstice, and we suggest that this could indicate a temporal effect, i.e. the production of winged individuals influenced by both generational and seasonal (temperature and humidity) effects (see Fig. 1). Evolving a response (for winged offspring production) to the environmental factors such as temperature or day length could enable aphids to synchronize their development with the optimal dispersal time and maximize their chance of finding new host plants [18]. Tansy aphids are non host-alternating species, therefore the produced winged morphs during the 3^rd^ to 5^th^ generations are mainly for migration to, and colonization of, new host plants.

### Winged Offspring Production by Winged and Unwinged Mothers

The production of winged individuals by winged mothers is uncommon, except during the last part of their reproductive lifespan [20]. We found that winged mothers in both aphid species produced winged offspring. In MA, the proportion of winged offspring was much higher for unwinged than winged mothers. The number of winged offspring produced by winged mothers was considerably larger in the greenhouse than the climate chamber experiment and may be due to the time in the season or plant quality. In contrast, approximately half of the winged ME mothers produced both winged and unwinged offspring. Thus, low production of winged offspring by winged mothers in MA is consistent with previous studies [16], [42]; however, for the ant-tended ME production of winged offspring is higher.

Because of lower reproduction of winged morphs, aphid clonal growth is higher if only unwinged offspring are produced, thus production of winged morphs should be limited to necessary dispersal events [43], [44]. MA aphids were found to produce winged offspring throughout the season, which may represent a more stable reproductive strategy for these aphids as they are not protected by ants from predation. This also may be a result of the effect of crowding, which we showed to influence winged morph production in MA. Once a winged morph has located a new host plant then it produces mainly unwinged offspring, thus enhancing colony growth. On the other hand, the ME aphids generally produced winged offspring only at the beginning of the season and therefore it may be beneficial for winged mothers to also produce winged offspring to maximize dispersal during this unique event.

### Effect of Crowding

Winged morph production and dispersal have both been considered as a driver of density regulation in aphids, and in many species the production of winged individuals is strongly density-dependent [15], [16], [19], [23]. Production of winged individuals among aphid populations in a density-dependent fashion is possibly the best strategy for maximizing the number of migrants produced during the life of a colony and seasonal cycle of a clone [18]. In the present study, our results clearly showed that MA is responsive to crowding (tactile stimulation) and produced more winged offspring when surrounded by conspecifics than when alone. MA aphids also produced fewer offspring in the crowding treatment, which may indicate increased stress levels; however, it is also a general phenomenon that aphids produce fewer winged than unwinged offspring, as these need more resources. Despite the clear effect of crowding for MA, the effect size was small in comparison with pea aphid which produces a much higher proportion of winged morphs in response to crowding [16].

We found no effect of crowding for the ant-tended ME on wing production or offspring number. If an aphid often experiences high densities, it may seem reasonable to assume that they would have a weaker response to crowding. However, numerous empirical studies showed that the crowding has considerable effect on wing induction in gregarious aphid species (often found in tight groups) such as *Aphis craccivora* [22], *Megoura viciae* [23] and *Rhopalosiphum padi* [45] while it has less effect on non-gregarious species like *Myzus persicae* [46]. Non-gregarious aphids that do not often come into contact with other aphids, possibly exhibit little response to crowding stimuli since this effect is absent in their natural habitat and thus no adaptation to it has occurred [47]. The reason for our ME results is therefore more likely to be the presence of mutualistic ants. A number of studies show that the presence of attending ants inhibits the production of winged individuals in *Aphis fabae* [48], [49], [50] and *Lachnus allegheniensis* attended by *Formica obscuripes* [51].

### Effect of Predators on Wing Induction

Our results clearly showed that for MA the presence of predators increased the production of winged offspring, which is consistent with studies on a number of other aphid species [25], [26], [28], [32]. MA aphids produced a greater proportion of winged offspring in the second period, and this delayed response could be because determination of offspring phenotype occurs some time before birth; thus, the first offspring born in the first period are likely to be determined before the experiment started.

We again found contrary results for the ant-tended ME compared to MA aphids, with no effect of predators on the proportion of winged offspring produced. In our study, a small number of ME aphids were consumed showing that the predators did attempt to feed but were not very successful due to the protective role of attending ants. In a different aphid species, Dixon & Agarwala [25] also found that ant-tended colonies of *Aphis fabae* did not respond to the presence of the predator by producing more winged offspring. However, in *Aphis gossypii,* which it also attended by ants, the presence of natural enemies was found to lead to an increase in the percentage of winged offspring [29]. For ant-attended aphids, it may not be beneficial to produce winged morphs in response to predator presence; most of the time the ants will protect the aphid and furthermore, when the winged individual leaves the natal plant it loses the advantages of protection by ants.

### Ecological Advantages of Winged Morph Production

The two main advantages of producing winged dispersal morphs in aphids are: 1) migration and dispersal between different host plants, and 2) escape from adverse environmental conditions. Dispersal between primary and secondary host plants during the season is fundamental for the survival of host-alternating aphid species (heteroecious). However, dispersal is also important for non host-alternating aphid species (autoecious), such as the tansy aphids studied here, because they allow aphids to disperse among several plants and enhance the reproductive chances. We generally found low proportions of winged offspring, which may be due to the tansy host species being a perennial plant. Previous work suggests that species feeding on perennial host plants, like tansy, exhibit a lower occurrence of winged morph production in comparison with those live on short persistence host plants, e.g. annual herbaceous plants [52]. Aphids can only exploit annual herbaceous plants, such as many crop species, for a short time period and therefore migration (by winged dispersal morphs) to new or more persistent hosts must take place at some point during the life cycle. Escape from adverse conditions such as decreasing plant quality, increasing interspecific competition caused by crowding and presence of natural enemies are important in order to maximize reproductive output [26], [43], [53].

The aphids we studied here exhibit classic metapopulation structuring in natural populations, and dispersal between plants is important for recolonization after the frequent extinction events that characterize such a system [37], [54]. Weisser [37] argued that in the tansy system the main driver of local population extinction is from natural enemies (parasitoids and predators). In this paper, we showed that MA aphids produce winged individuals throughout the season and respond to predator attack by producing more winged individuals. This indicates they have evolved these traits to enable escape from areas of high predation pressure, and enhance recolonization over the whole season. The second aphid species we studied (ME) does not produce winged offspring throughout the season, neither does it respond to predation, and this is likely related to the aphid-ant mutualism they have evolved where the ant protects the aphids from predation and the aphid produces honeydew to feed the ants. These aphids are obligate mutualists and without ant-attendance they rarely survive [55], therefore any selective force for increased wing production due to predation pressure will be reduced.

In conclusion, aphids respond to various stimuli for the production of winged morphs, which will help them to track environmental conditions much more reliably [18]. We found that the cues that induced winged morph production varied among aphid species, likely due to whether they were ant-tended or not, and included temporal effects, maternal effects, crowding and presence of a predator. Understanding the role of environmental cues for wing induction in aphids in a metacommunity system will benefit the study of life-history evolution in spatially heterogeneous habitats.

## Supporting Information

Figure S1 Illustration of the transferring of aphids to new plants in the effect of predators on wing induction experiment. The flow chart shows the experimental design for one aphid line and was the same for all aphid lines.
(TIF)

Figure S2 Photograph of *Macrosiphoniella tanacetaria* sexual female (Oviparae) with its egg. The sexual morphs of this aphid species, produced in the autumn, lay overwintering eggs after mating.
(TIF)
