# Supplementary figures and images for: Multiple Cues for Winged Morph Production in an Aphid Metacommunity
Source: PLoS One. 2013 Mar 5;8(3):e58323. doi: 10.1371/journal.pone.0058323 (PMC3589340; doi:10.1371/journal.pone.0058323)

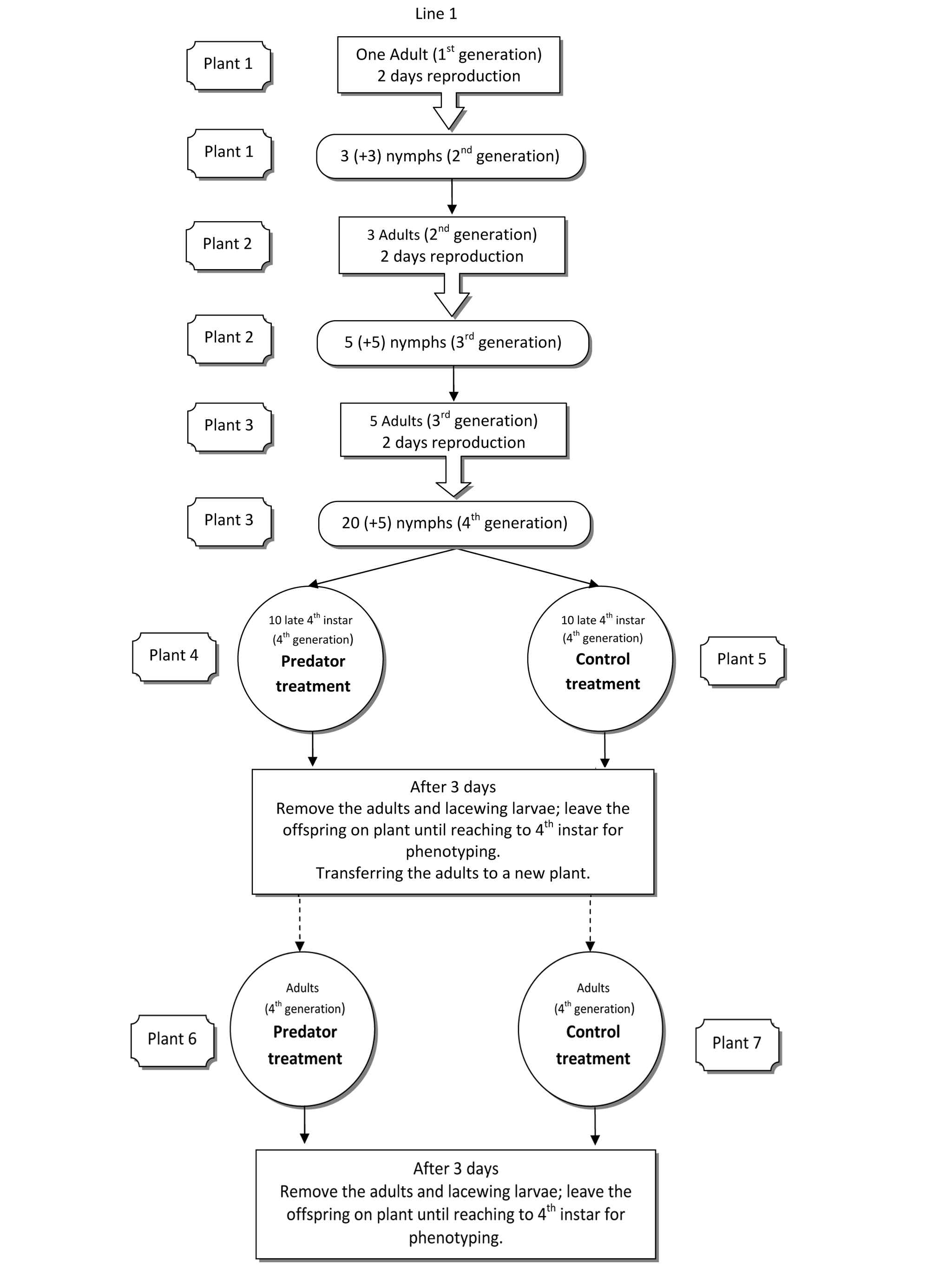

Supplement: Figure S1 — Illustration of the transferring of aphids to new plants in the effect of predators on wing induction experiment. The flow chart shows the experimental design for one aphid line and was the same for all aphid lines. (TIF) [file pone.0058323.s001.tif]

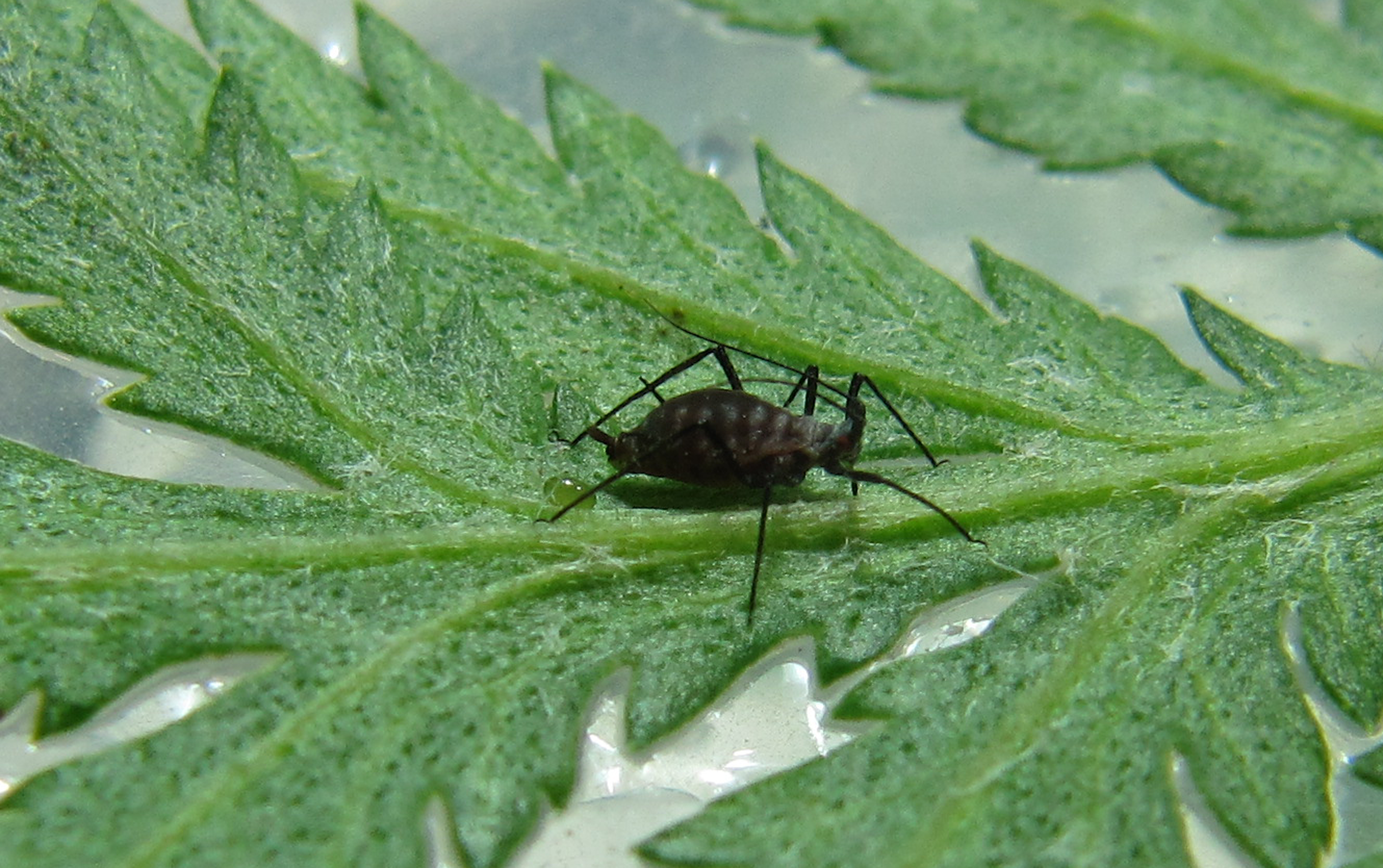

Supplement: Figure S2 — Photograph of Macrosiphoniella tanacetaria sexual female (Oviparae) with its egg. The sexual morphs of this aphid species, produced in the autumn, lay overwintering eggs after mating. (TIF) [file pone.0058323.s002.tif]
